# Chondrosarcoma organoids reveal SHH pathway activation driven by *PTCH1* and *BCOR* alterations

**DOI:** 10.1038/s41598-025-33061-z

**Published:** 2025-12-19

**Authors:** Haruna Takami, Keiichi Yoshida, Yukiko Matsuoka, Satoru Sasagawa, Noriko Nagamine, Yoji Kukita, Kazuma Kiyotani, Yusuke Yoshimura, Rie Suzuki, Hironari Tamiya, Shigeki Kakunaga, Toshinari Yagi, Sho Nakai, Yoshinori Imura, Seiji Okada, Ken-ichi Yoshida, Satoshi Takenaka, Toru Wakamatsu

**Affiliations:** 1 Department of Musculoskeletal Oncology Service, Osaka International Cancer Institute, 3-1-69 Otemae, Chuo-ku, Osaka, 541-8567 Japan; 2 Department of Orthopaedic Surgery, Osaka University Graduate School of Medicine, 2-2 Yamadaoka, Suita, Osaka, 565-0871 Japan; 3 Next-generation Precision Medicine Research Center, Osaka International Cancer Institute, 3-1-69 Otemae, Chuo-ku, Osaka, 541-8567 Japan; 4 Molecular Biology Laboratory, Research Institute, Nozaki Tokushukai Hospital, Tanigawa 2-10-50, Daito, Osaka, 574-0074 Japan; 5 Laboratory of Genomic Pathology, Osaka International Cancer Institute, 3- 1-69 Otemae, Chuo-ku, Osaka, 541-8567 Japan; 6 Laboratory of Immunogenomics, Center for Intractable Diseases and ImmunoGenomics (CiDIG), Health and Nutrition (NIBIOHN), National Institute of Biomedical Innovation, Ibaraki-shi, Osaka, Japan; 7 Department of Diagnostic Pathology and Cytology, Osaka International Cancer Institute, 3-1-69 Otemae, Chuo-ku, Osaka, 541-8567 Japan

**Keywords:** Chondrosarcoma, Organoid, Sonic hedgehog, *PTCH1*, *BCOR*, Vismodegib, Cancer, Oncology

## Abstract

Chondrosarcoma is the second most common malignant bone tumor, characterized by the production of cartilaginous matrix and a high degree of resistance to conventional therapies such as chemotherapy and radiotherapy. Effective treatment options remain limited, highlighting the urgent need for preclinical models to explore novel therapeutic approaches. This study aimed to establish patient-derived organoid (PDO) models of chondrosarcoma and to investigate their utility in elucidating molecular mechanisms and drug responses. Chondrosarcoma specimens were collected from patients and cultured using a modified air–liquid interface (ALI) organoid method. The resulting PDO were serially expanded *in vitro* and transplanted into NOD-SCID IL2Rgnull mice for *in vivo* validation. Histological and genetic analyses were performed to compare organoids with the corresponding primary tumors. Whole-exome profiling was used to identify genetic alterations. Organoid-based drug sensitivity testing was conducted using vismodegib, a Sonic Hedgehog (SHH) pathway inhibitor. Two PDO lines were successfully established. Organoid-derived xenografts preserved the histological and genetic features of the parental tumors. Genomic profiling revealed loss-of-function mutations in *PTCH1* and *BCOR*, suggesting activation of the Sonic Hedgehog signaling pathway. Consistently, vismodegib exhibited strong *in vitro* antitumor activity, indicating functional pathway dependence. We established the first PDO models of chondrosarcoma that faithfully recapitulate key tumor features. These models provide a valuable preclinical platform for dissecting molecular pathogenesis and for advancing the development of targeted therapeutic strategies in this intractable malignancy.

**Supplementary Information:**

The online version contains supplementary material available at 10.1038/s41598-025-33061-z.

## Introduction

Chondrosarcoma (CS) is the second most prevalent malignant bone tumor and is distinguished by the formation of a cartilaginous matrix^1–3^. CS predominantly affects middle-aged individuals in their 50 s and is classified as a high-grade malignancy with a high risk of local recurrence and distant metastasis^1,4–8^. Although complete surgical resection remains the prevailing treatment, effective chemotherapeutic options are lacking for advanced-stage CS.

The 2020 WHO classification system is predicated on the stratification of CS based on histological grade and anatomical location^9–11^. Low-grade lesions in the appendicular skeleton are designated as atypical cartilaginous tumors (ACT), whereas similar histology in the axial skeleton is classified as grade 1 CS. High-grade CS encompasses grade 2–3 conventional types as well as dedifferentiated, mesenchymal, and clear cell subtypes^4,12^.

Recent genomic studies have revealed recurrent mutations in genes such as *IDH1*, *IDH2*, and *COL2A1*, particularly in central conventional and dedifferentiated CS^13–17^. Among these, *IDH1* mutations are regarded as potential therapeutic targets. Preliminary clinical trials employing isocitrate dehydrogenase inhibitors, including ivosidenib, have demonstrated encouraging efficacy^18–22^. However, the clinical application of these findings remains constrained due to the rarity of the disease and limited availability of reliable preclinical models.

Organoid culture systems have emerged as a promising platform for modeling rare cancers, including sarcomas^23–25^. These systems offer the potential to facilitate patient-specific drug testing and molecular characterization, which could lead to more precise and targeted treatment options for patients with these rare cancers. While organoid technology has been widely applied in the study of epithelial tumors, its adaptation to mesenchymal malignancies, including CS, is still in its infancy^24–26^. The present undertaking entails the establishment of patient-derived organoid (PDO) lines from multiple sarcoma subtypes, with the objective of developing a robust preclinical platform based on these models^27–31^. Robust PDO models of CS have the potential to serve as valuable tools for mechanistic studies and to accelerate the development of targeted therapies for this intractable disease.

In this study, we established PDO models of CS and evaluated their histological, molecular, and pharmacological characteristics to assess their potential as preclinical models.

## Results

### Establishment of PDO models from human patients with CS

**Fig. 1:**
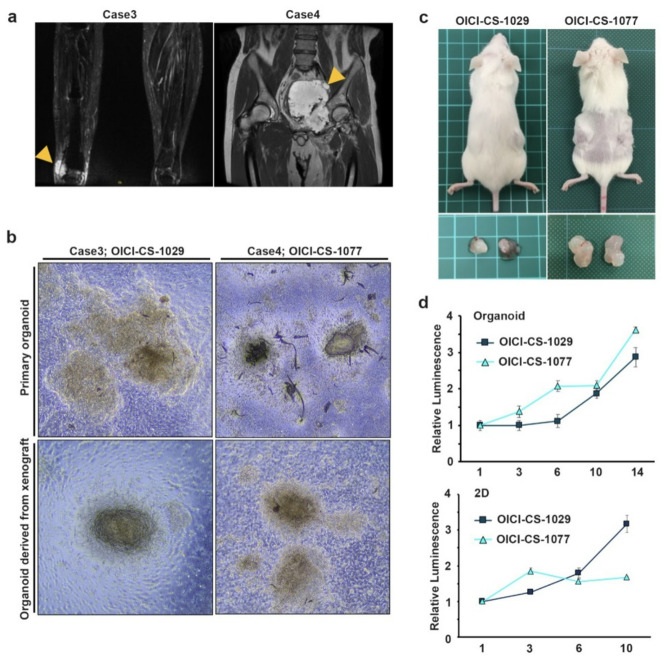
The establishment of PDO and ODX models from patients with CS. (**a**) The clinical images depict the CS tumors in the right fibula (case 3) and pelvis (case 4). The coronal short tau inversion recovery (STIR) image of the magnetic resonance imaging (MRI) was shown in the left panel. Right: The following is a T2-weighted coronal image of the MRI. The tumor was indicated by yellow triangles. (**b**) The images of OICI-CS-1029 and OICI-CS-1077 organoids under ALI organoid method using phase-contrast microscopy. The morphology of organoids from both the primary culture and the xenograft-derived culture was demonstrated. (**c**) The development of tumors in NSG mice of ODX from the PDO of CS was observed. (**d**) The relative cell viability of the CS in organoids or adherent cultures was measured from day 1 to 10. STIR, short tau inversion recovery; MRI, Magnetic Resonance Imaging; ALI, air–liquid interface; ODX, organoid-derived xenograft; PDO, patient-derived organoids; NSG, NOD/SCID/IL2Rγnull; CS, chondrosarcoma.

Of six CS cases examined, four generated primary air–liquid interface (ALI) organoid cultures with tumorigenic capacity when engrafted into mice. The clinical and experimental information of the examined cases is summarized in Table 1, while detailed clinical courses are provided in Supplementary Fig. 1. However, one case (case 1; OICI-CS-0934) successfully formed tumors in mice (0.6, 1.2 cm) but exhibited substandard *in vitro* proliferation and could not be sustained in long-term culture (Supplementary Fig. 2). Another case (case 6; OICI-CS-1329) yielded a stable PDO line; however, phenotypic and molecular characterization has yet to be performed. The present study centered on two CS PDO lines, designated as OICI-CS-1029 and OICI-CS-1077, that had been successfully established and characterized (Fig. 1a, b, and c). The cells demonstrated stable proliferation over time in serial passages. The PDO demonstrated an irregular spherical morphology in the primary culture, a feature that was largely retained during subsequent passages. Despite the presence of scattered peripheral cells, spindle-shaped cells were seldom detected (Fig. 1b). *In vivo* tumorigenicity was confirmed for both CS PDO lines, which formed subcutaneous tumors measuring approximately 1 cm in size over a period of more than 8 months (Fig. 1c). The tumor sizes were as follows (maximum diameter): OICI-CS-1029 (0.85, 1.1 cm), OICI-CS-1077 (1.4, 1.4 cm). The formed tumors exhibited a translucent appearance, and their gross morphology suggested the presence of cartilaginous tissue (Fig. 1c, bottom). The PDO demonstrated a two-fold increase in viability within approximately 10 days under *in vitro* culture conditions, indicating their potential utility for downstream experimental applications (Fig. 1d, upper). In collagen-coated adherent conditions, the OICI-CS-1029 cells demonstrated a three-fold increase over a 10-day period. Conversely, the OICI-CS-1077 cells exhibited minimal proliferation between days 3 and 6 (Fig. 1d bottom).

**Table 1:**
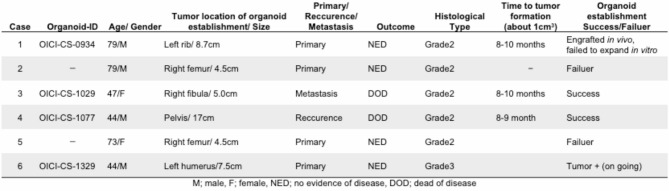
The clinical information of patients with CS, as well as the detailed ODX data, are presented herein. CS, chondrosarcoma; ODX, organoid-derived xenograft.

### Morphologic characteristics of PDO of CS

**Fig. 2:**
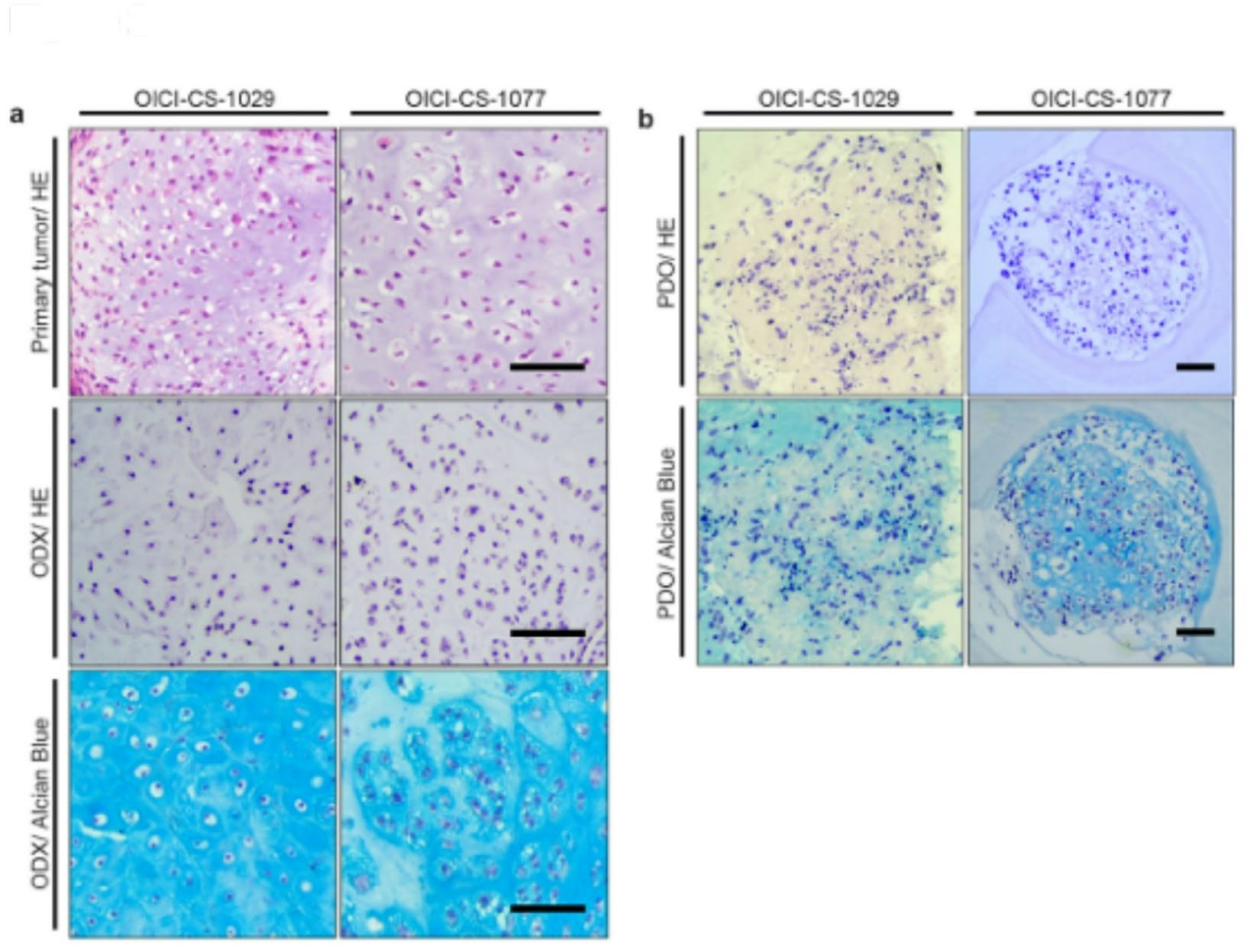
A comparison of the microscopic features of the patient’s original tumors was conducted, as well as an analysis of ODX and PDO of CS models. (**a**) Histological appearance of the original tumors and ODX of OICI-CS-1029 and OICI-CS-1077 with hematoxylin and eosin (H&E) staining (upper and middle) and Alcian Blue staining (lower). (**b**) Histological appearance of the PDO of CS models with H&E staining (upper) and Alcian Blue staining (lower). Scale bars: 100 μm. ODX, organoid-derived xenograft; PDO, patient-derived organoids; CS, chondrosarcoma.

A histological examination of the original CS tumors revealed abundant hyaline cartilage matrix and lobulated growth patterns in both cases (Fig. 2a top). Furthermore, both tumors exhibited moderate nuclear atypia and increased cellularity, which is consistent with a pathological diagnosis of grade 2 CS. These characteristics were retained in the tumors that developed following transplantation of the PDO into mice (Fig. 2a middle, Supplementary Fig. 2 d). In order to assess matrix composition, Alcian Blue staining was applied to the xenograft tumors, which confirmed the presence of cartilaginous matrix (Fig. 2a bottom and Supplementary Fig. 2 d). Furthermore, the presence of cartilaginous matrix was also confirmed in the organoids under *in vitro* culture conditions (Fig. 2b). These findings indicate that the two established PDO lines of CS recapitulate the characteristic histological features of CS.

### Genetic characteristics of PDO models of CS

**Fig. 3:**
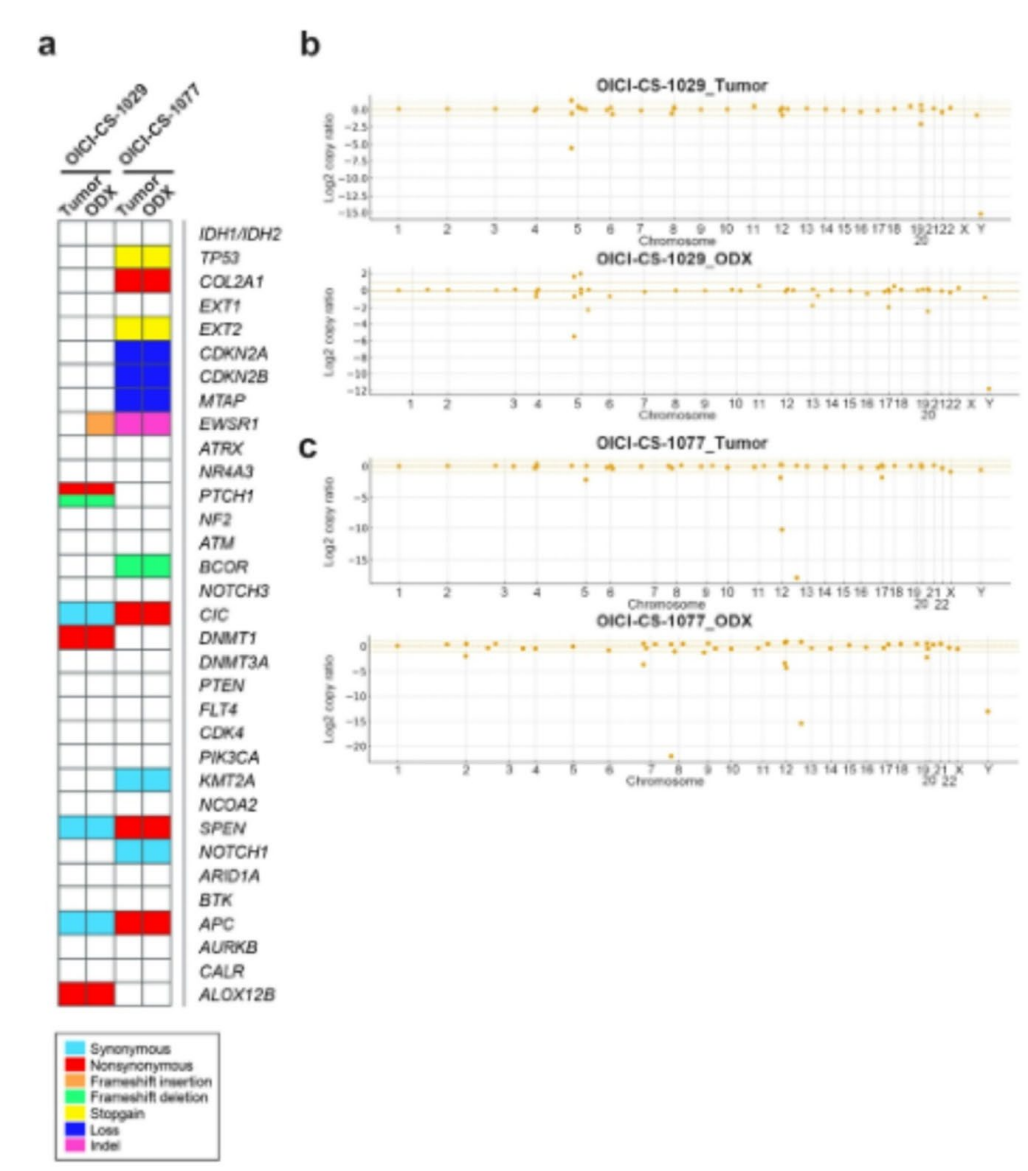
Genetic characteristics in the primary tumors and in the CS models of OICI-CS-1029 and OICI-CS-1077. (**a)** Somatic mutations, including single-nucleotide variants (SNV), short insertions/deletions (INDELs), and copy number variations (CNV), were demonstrated. (**b**) CNV profiles of the original tumors and the corresponding ODX of OICI-CS-1029 were then visualized using chromosomal plots. (**c**) CNV profiles of the original tumors and the corresponding ODX of OICI-CS-1077 were then visualized using chromosomal plots. SNV, single-nucleotide variant; INDEL, short insertions/deletions; CNV, copy number variation.

Whole-exome sequencing (WES) was performed to identify genetic alterations in the PDO models of CS. A high proportion of the genetic alterations found in their corresponding primary tumors was retained by both CS organoid-derived xenografts (ODX) (Fig. 3a). Subsequent analysis of the CS ODX revealed the absence of mutations in *IDH1* or *IDH2*. Frameshift mutations resulting in truncation were identified in *PTCH1* and *BCOR* in the 1029 and 1077 cells, respectively, as potential driver alterations (Fig. 3a). Furthermore, the OICI-CS-1077 ODX harbored well-characterized pathogenic mutations in *TP53*, *COL2A1*, *CDKN2A*, *CDKN2B*, and *MTAP*, all of which have been previously implicated in the pathogenesis of CS (Fig. 3a)^13–17^. Furthermore, OICI-CS-1029 ODX exhibited a mutation in *DNMT1*, which encodes a DNA methyltransferase that plays a crucial role in the maintenance of DNA methylation patterns (Fig. 3a)^30–32^. A mutation in *EXT2*, a gene known to be involved in osteochondroma development, was identified in OICI-CS-1077 ODX (Fig. 3a)^33,34^. Copy number variation analysis revealed that both chondrosarcoma ODX maintained the genomic alterations characteristic of their parental tumors, albeit to a limited extent (Figs. 3b and c). These findings suggest that the established chondrosarcoma PDO models could recapitulate the identified genetic alterations to a certain extent, and that these alterations may potentially play a critical role in the tumorigenesis of these CS.

**Fig. 4:**
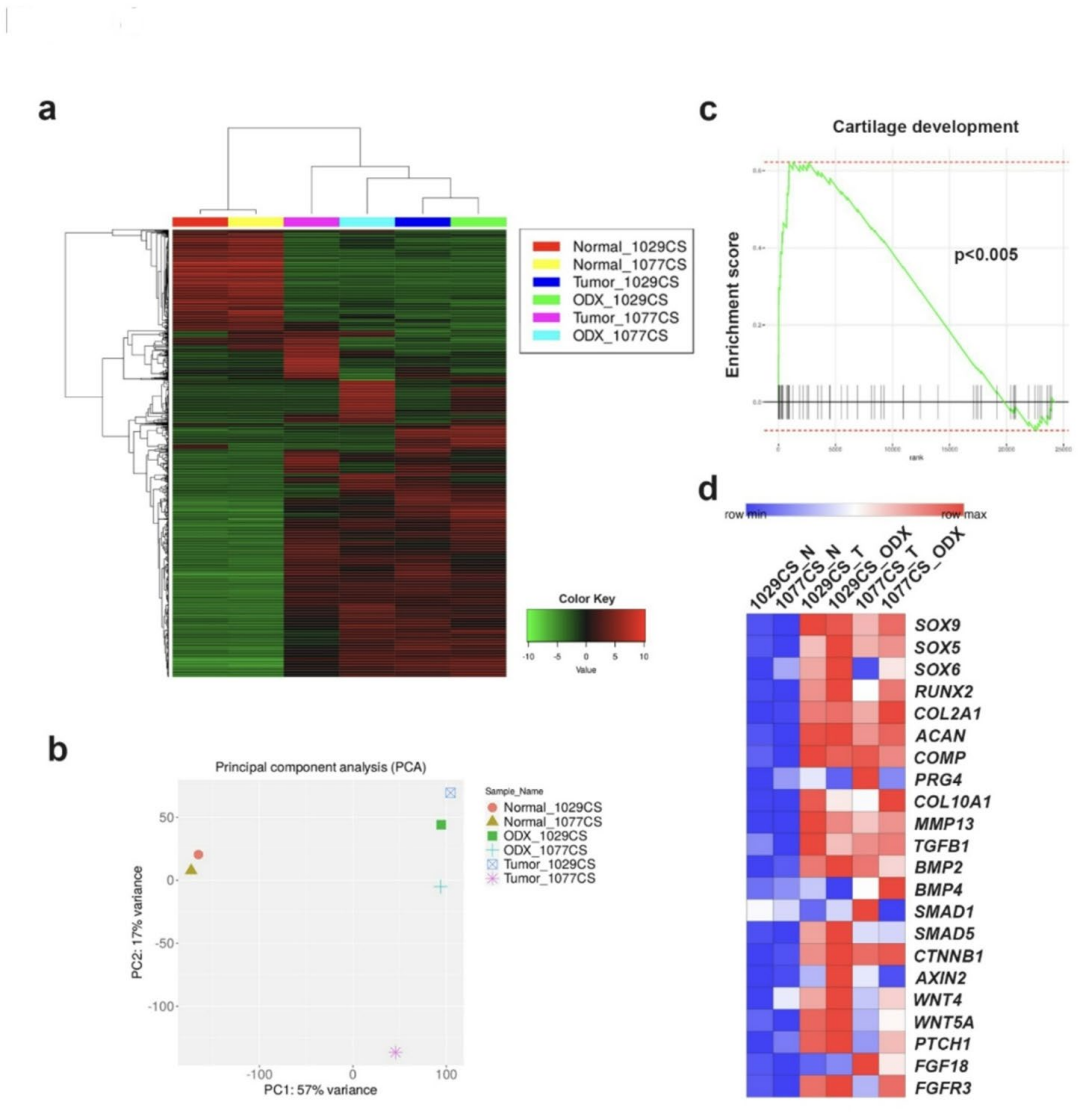
A comparison of the characteristics of ODX of CS and original tumors was conducted through a comprehensive analysis of the respective transcriptomes. (**a**) Heatmap illustrating the gene expression profiling (1000 genes) of patient-matched adjacent skeletal muscle tissues collected at surgery (Normal), original CS tumors (Tumor), and CS ODX tumors developed in NSG mice. (**b**) The following investigation was conducted using principal component analysis (PCA) to compare the normal, tumor, and ODX of OICI-CS-1029 and OICI-CS-1077. (**c**) The following investigation was conducted: a gene set enrichment analysis (GSEA) of the genes related to cartilage development was performed on the ODX models of CS. (**d**) Expression levels of genes commonly associated with chondrogenesis were extracted from RNA sequencing data. ODX, organoid-derived xenograft; CS, chondrosarcoma; PCA, principal component analysis; GSEA, gene set enrichment analysis.

To further characterize the PDO models of CS, RNA sequencing was performed to investigate their transcriptomic landscape. Hierarchical clustering analysis revealed that each ODX was classified in proximity to its corresponding primary tumor (Fig. 4a). In the context of principal component analysis (PCA), the OICI-CS-1029 ODX demonstrated a notable degree of similarity to its tumor of origin, while the OICI-CS-1077 ODX exhibited a moderate level of similarity (Fig. 4b). Furthermore, scatter plot analysis revealed a high degree of transcriptomic correlation between the ODX and their corresponding primary tumors, with R values of 0.90 for OICI-CS-1029 and 0.84 for OICI-CS-1077 (Supplementary Fig. 3). These findings support the hypothesis that the established PDO models of CS closely reflect the gene expression landscapes of their respective original tumors. In accordance with these findings, gene set enrichment analysis (GSEA) of chondrogenesis-related genes revealed that these signatures were also significantly enriched in the CS ODX (Fig. 4c). Genes associated with chondrogenic differentiation, including *SOX9*, *RUNX2*, *ACAN*, and *COL2A1*, as well as other indicators of this differentiation, demonstrated heightened expression levels in both the initial CS tumors and the corresponding ODX (Fig. 4d). These findings further support the hypothesis that the PDO models of CS recapitulate the chondrogenic transcriptional profile of their primary tumors.

### The Sonic Hedgehog (SHH) signaling pathway represents a potential therapeutic target in PDO models of CS

**Fig. 5:**
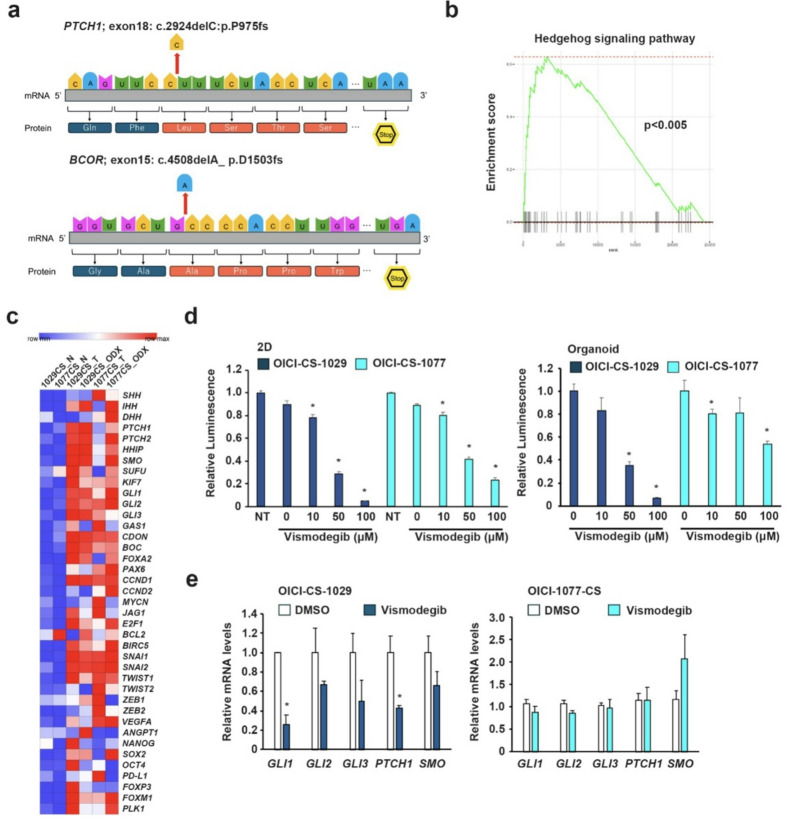
Experiments were conducted to ascertain the efficacy of pharmaceutical agents when applied to CS organoids, with a particular focus on their ability to counteract the effects of SHH inhibitors. (**a**) Schematic representation of the frameshift mutation detected in the *PTCH1* and *BCOR* gene, highlighting the disrupted coding sequence. (**b**) The following investigation will involve the GSEA of hedgehog signaling pathway-related genes of ODX models of CS. (**c**) Expression levels of SHH-related genes based on RNA-seq data were compared among normal tissues, the original CS tumors, and the ODX. The results of the study were then transformed into a heatmap for further analysis. (**d**) Cell viability of CS in adherent or organoid culture treated with vismodegib for three or seven days, respectively (*n* = 3; *P < 0.05). (**e**) The following comparison was made: the levels of gene expression related to the SHH signaling pathways were compared using qPCR in CS organoids treated with vismodegib (100 µM) for two days. SHH, Sonic Hedgehog; ODX, organoid-derived xenograft; CS, chondrosarcoma; GSEA, gene set enrichment analysis; qPCR, quantitative polymerase chain reaction.

To investigate potential therapeutic targets in CS, the focus was directed towards a frameshift mutation in *PTCH1* and *BCOR*. It has been established that loss-of-function mutations in *PTCH1* result in constitutive activation of the SHH signaling pathway^35–37^. A *PTCH1* frameshift mutation was identified in the OICI-CS-1029 ODX, resulting in a truncation that confers a loss-of-function alteration (Fig. 5a). A *BCOR* frameshift mutation detected in OICI-CS-1077 has also been implicated as a potential mechanism underlying SHH pathway activation in other types of cancer^38,39^. GSEA revealed significant enrichment of the SHH signaling pathway. As illustrated in Figs. 5b and c, a significant number of SHH-related genes were found to be overexpressed in both CS tumors and their corresponding ODX. Consequently, the present study hypothesized that the SHH pathway could serve as a therapeutic target. Therefore, an investigation was conducted to determine whether vismodegib, an SHH inhibitor, could suppress the growth of CS organoids. In the OICI-1029-CS cells, vismodegib significantly suppressed cell proliferation under both adherent and organoid culture conditions (Fig. 5d). In contrast, the OICI-1077-CS cells also exhibited growth inhibition under both conditions, but the effect was less pronounced compared with that observed in the OICI-CS-1029 (Fig. 5d). The inhibitory effect of vismodegib on SHH signaling was validated using quantitative PCR (qPCR). In OICI-CS-1029, SHH-related gene expression was significantly suppressed, while no suppression was observed in OICI-CS-1077 (Fig. 5e). A collective examination of the findings indicated the potential of the SHH pathway as a therapeutic target in a specific subset of cases of CS.

## Discussion

This study demonstrated the potential efficacy of SHH pathway inhibition in a subset of CS. A body of research has previously indicated that there is aberrant activation of the SHH signaling pathway in cases of CS, which suggests the potential for therapeutic interventions. In fact, Campbell et al. demonstrated that the Smoothened (SMO) inhibitor IPI-926 suppressed tumor growth and downregulated Hedgehog target genes in xenograft models of CS^40^. Conversely, Wang et al. later reported that SMO inhibition alone had limited effects in cell models due to non-canonical *GLI1* activation^41^. However, to date, there has been a paucity of direct evidence for the efficacy of SHH pathway inhibitors in patient-derived models. The present findings extend these observations by demonstrating differential sensitivity to vismodegib in CS organoids and thereby support the notion that SHH pathway inhibition may benefit a subset of patients.

Ivosidenib, an IDH1 inhibitor, is currently being investigated as a potential therapeutic option for CS with *IDH1* mutations. The efficacy of this agent has been assessed in clinical trials for cholangiocarcinoma, a neoplasm that is distinguished by a relatively high frequency of *IDH1* mutations^21,22^. In the PDO models of CS established in this study, no *IDH1* mutations were identified, precluding an assessment of the efficacy of ivosidenib. Nevertheless, further establishment of organoid models may enable such investigations and support the development of mutation-guided therapeutic strategies. Given that ivosidenib has already received regulatory approval for *IDH1*-mutant cholangiocarcinoma, the evaluation of this agent in *IDH1*-mutant CS models would be of particular translational relevance once such models are established.

Subsequent analysis focused on frameshift mutations in *PTCH1* and *BCOR* as potential mechanisms underlying SHH pathway activation^40–44^. As demonstrated in the research, loss-of-function mutations in *PTCH1* have been identified as a primary cause of constitutive SHH pathway activation, particularly in colorectal and gastric cancer^42,43^. Furthermore, BCOR functions as a tumor suppressor within the PRC1.1 complex, and its inactivation accelerates SHH-driven tumorigenesis in medulloblastoma models^38^. The findings of this study provide a foundation for further research, suggesting that the frameshift mutations identified in the CS organoids may contribute to pathway activation. However, to the best of our knowledge, SHH activation driven by these mutations has not been directly examined in CS. We endeavored to induce the expression of *PTCH1* and *BCOR* through lentiviral transduction and chemical transfection. However, these experiments proved unsuccessful due to suboptimal transduction efficiency and cellular toxicity. These limitations are likely attributable to the technical constraints inherent to the organoid system. However, as more cases are accumulated, it may become possible to validate the causal role of these mutations in SHH pathway activation in CS.

Frameshift or indel alterations in *EWSR1* were also detected in both PDO lines. Although *EWSR1* alterations are well-established oncogenic drivers in specific sarcoma subtypes, their role in CS is currently unclear. It is possible that disruption of *EWSR1* may contribute to genomic instability or clonal dynamics, particularly in OICI-CS-1077, which exhibited more extensive copy-number alterations. Further functional studies will be necessary to determine whether *EWSR1* loss influences tumor evolution or SHH pathway dependence in CS.

The present study has several limitations. Both PDO lines in this study were established from patients with advanced-stage CS with poor clinical outcomes. This raises the possibility that aggressive disease biology and high tumor burden may favor successful PDO and ODX establishment, potentially due to enrichment of proliferative or stem-like tumor cell populations. However, because these PDO were derived from high-grade cases, they may predominantly reflect aggressive disease biology and may not fully represent the broader spectrum of lower-grade or early-stage CS. Future studies should include PDO derived from a wider range of pathological grades and clinical stages to clarify how organoid establishment success correlates with clinical and pathological features.

Initially, the PDO models were established at a single institution using a modified ALI method. The reproducibility of these organoids across sarcoma subtypes and laboratories remains to be determined. Secondly, the number of samples from CS patients was limited, which necessitates validation of the SHH pathway dependency in larger cohorts for generalizability. Thirdly, the technical challenges intrinsic to organoid models, including the low efficiency of genetic manipulation and the cellular toxicity associated with transduction or transfection, constrained our capacity to functionally validate the impact of specific mutations.

In addition, although early-stage organoids (within approximately 10 days of in vitro culture) are technically adequate for genomic analyses, we found that these cultures often exhibit unstable growth and a progressive increase in stromal components. Mouse xenograft passaging was not required for organoid formation itself, but it markedly improved biological stability by enriching tumor-propagating cells and reducing non-malignant tissue contamination. As a result, ODX-derived PDO provided clearer tumor-specific molecular signatures and greater robustness for downstream applications, including therapeutic screening. These findings suggest that while early organoids can be used for mutation detection, ODX-stabilized models offer superior biological fidelity for translational research.

Furthermore, although PDO may serve as an alternative preclinical drug-testing platform, we did not perform *in vivo* drug treatment using the corresponding ODX models. CS exhibits very slow tumor growth *in vivo*, requiring a prolonged duration to achieve measurable tumor size suitable for therapeutic evaluation. Therefore, ODX-based drug efficacy testing was not feasible within the scope of this exploratory PDO establishment study. Future studies will focus on optimizing experimental conditions to improve tumor growth kinetics and integrating PDO-based drug screening with ODX-based validation to strengthen the clinical translatability of PDO-guided therapeutic strategies in CS. Finally, potential procedural biases associated with the single-center design cannot be completely excluded.

In summary, this study presents, to the best of our knowledge, one of the initial human organoid models of CS. This finding suggests that the SHH signaling pathway may serve as a viable therapeutic target in a subset of cases. These models offer insight into the molecular pathogenesis of the disease and establish a preclinical platform for mutation-guided therapeutic development that may ultimately improve patient outcomes.

## Methods

### Patient

During the 2022–2024 period, our hospital provided treatment to a total of six patients diagnosed with CS. The patient population was exclusively Japanese, comprising four male and two female individuals. The subjects were between 40 and 80 years of age at the time of tumor sample collection. An attempt was made to establish PDO from residual tumor samples obtained during surgery. The clinical information and detailed ODX data were summarized in Table 1, and the clinical courses were described in Supplementary Fig. S1. Informed consent was obtained from all participants in the study. The study was approved by the Ethics Committee of Osaka International Cancer Institute (approval number: 1710059174-12), and all procedures were conducted in accordance with institutional and national ethical guidelines.

### PDO and ODX establishment

We selected the ALI method based on our prior experience and initial trials, in which it provided more reliable early organoid formation from sarcoma tissues compared with conventional submerged cultures^44,45^. Through these preliminary experiences, ALI method became the preferred approach in our laboratory for establishing CS PDO. Fresh CS tumor tissues were collected from surgical specimens and processed as previously described^44–46^. The cultivation of tumor cells was accomplished through the implementation of the ALI organoid method. The basal medium used for organoid culture consisted of Advanced DMEM/F12 (Thermo Fisher Scientific, Waltham, MA, USA) with the following supplements: 10 mM HEPES, 1× GlutaMAX, and 1× penicillin-streptomycin-glutamine (also from Thermo Fisher Scientific). Furthermore, StemFit AK02N (Ajinomoto Healthy Supply, Tokyo, Japan) was utilized for the purpose of organoid culture, in accordance with the manufacturer’s guidelines. Following the completion of several passages, the organoids were xenografted into six-week-old NOD-SCID IL2Rgnull (NSG) mice. NSG mice were purchased from Oriental Yeast Co. Ltd. (Tokyo, Japan). Tumor outgrowths were harvested, processed, and subsequently established in ALI cultures. This cycle of culture and xenograft passaging was repeated to establish stable PDO and ODX.

For each PDO line, one NSG mouse was used, and organoid fragments were implanted into two independent subcutaneous dorsal sites. Both OICI-CS-1029 and OICI-CS-1077 generated tumors at all injection sites, confirming consistent tumorigenic capacity. OICI-CS-0934 also produced small subcutaneous nodules, but these failed to progress and the line could not be propagated further. Due to the intrinsically slow *in vivo* growth of CS, xenograft passaging was performed up to the third generation, which represented the feasible experimental limit within the study period.

Mice were anesthetized with isoflurane and euthanized by cervical dislocation under deep anesthesia. Tumor dimensions (length and width) were measured using calipers at each observation time point. Animal condition and well-being were carefully monitored throughout the experiment, and body weights were measured when necessary, in accordance with institutional animal care and welfare guidelines. No apparent signs of illness or health deterioration were observed in any of the animals during the study. All procedures were performed in compliance with institutional animal care guidelines. The study was conducted in compliance with the ARRIVE guidelines. The animal experiment was approved by the Animal Experiment Committee of Osaka International Cancer Institute (approval number: 25030321), and all procedures were carried out in accordance with the relevant guidelines and regulations.

### DNA and RNA isolation

The isolation of genomic DNA was conducted using the DNeasy Blood & Tissue Kit (Qiagen), with adherence to the manufacturer’s instructions. Total RNA was purified using the RNeasy Plus Universal Mini Kit (Qiagen), and the quality of the RNA was assessed by measuring the A260/A280 ratio. Subsequently, 1 µg of RNA was reverse transcribed into cDNA using the SuperScript IV VILO Master Mix (Invitrogen).

### Hematoxylin and Eosin staining

The histological similarity between patient tumors and the corresponding PDO or ODX was assessed by processing tissue samples for routine hematoxylin and eosin (H&E) staining. Organoid and tumor specimens were fixed in 10% neutral-buffered formalin, embedded in paraffin, cut into 4-µm sections, and subsequently stained with hematoxylin and eosin according to standard protocols.

### Alcian blue staining

The deparaffinization and rehydration of the paraffin-embedded tissue sections were conducted using distilled water. Subsequently, the slides were immersed in acetic acid for a period of three minutes, followed by incubation in Alcian Blue staining solution for a duration of 30 min at room temperature. Following the staining process, the sections were rinsed with acetic acid to ensure the removal of excess dye. Subsequently, they were washed in running tap water for two minutes, accompanied by two additional water changes. The slides were then counterstained with hematoxylin, rinsed again in running tap water for two minutes with two changes, and dehydrated through a graded ethanol series. Subsequently, the sections were cleared and mounted for microscopic evaluation.

### Quantitative RT-PCR analysis

Quantitative RT-PCR was performed using TB Green Premix Ex Taq II (Takara Bio, Shiga, Japan) and gene-specific primers on the CFX96 Touch Real-Time PCR System (Bio-Rad, Hercules, CA, USA). The primer sequences (Supplemental Table S1) were obtained from PrimerBank (https://pga.mgh.harvard.edu/primerbank/). Normalization of expression levels was conducted relative to *ACTB*, designated as the internal reference gene.

### Proliferation assays

Adherent culture: Cultured cell suspensions (500 cells/well) were seeded onto collagen-coated 96-well plates (Iwaki, Tokyo, Japan) and subsequently incubated under standard conditions. After 24 h, the culture medium was replaced with a medium containing vismodegib. Following a three-day treatment period, cell viability was assessed using CellTiter-Glo^®^ 3D (Promega, Madison, WI, USA), and RNA was extracted for expression analysis.

Organoid culture: Organoids or xenograft-derived cells were embedded in collagen gel and cultured on Millicell^®^ inserts placed in 24-well plates. A bottom layer of 150 µL of collagen gel was allowed to solidify, and an upper layer containing cells in organoid culture medium was added. Cultures were maintained at 37 °C in a humidified 5% CO₂ atmosphere. Subsequent to a 72-hour period, treatment with vismodegib or verteporfin was initiated, and viability was assessed on days 3 or 7 using CellTiter-Glo^®^ 3D (Promega, USA).

### Whole-exome sequencing analysis

Genomic DNA was isolated from the tumors and matched normal tissues and subjected to WES outsourced to Rhelixa (Tokyo, Japan). The quality and concentration of the DNA were evaluated using a Qubit fluorometer and agarose gel electrophoresis. Exome libraries were prepared using the Agilent SureSelect Human All Exon V6 kit (58 Mb capture size) and sequenced on the Illumina NovaSeq 6000 platform. Initially, the raw reads were examined using the FastQC tool. Subsequently, they were processed with Trimmomatic (ILLUMINACLIP: 2:30:10) to remove adapter sequences and low-quality bases. The cleaned reads were then aligned to the GRCh38/hg38 reference genome using BWA-MEM, and PCR duplicates were removed with Picard. The base quality score recalibration was executed using the GATK BaseRecalibrator/ApplyBQSR pipeline. Somatic single nucleotide variants (SNVs) and small insertions/deletions were identified through the use of GATK Mutect2, structural variants were detected using Manta, and copy number alterations were characterized by means of CNVkit. Variant annotation was performed using OncoKB for clinical and biological interpretation. To further concentrate on genetic alterations relevant to chondrosarcoma, data for genes reported as recurrently mutated in the January 2022 version of My Cancer Genome was extracted and visualized.

### RNA sequencing and transcriptome analysis

Normal control tissues used for the transcriptomic analyses consisted of patient-matched adjacent skeletal muscle collected during wide resection surgery. A small portion of surrounding muscle without visible tumor infiltration was immediately snap-frozen and stored at −80 °C until RNA extraction. Total RNA from tumors, ODX, and matched normal tissues was extracted using the method described above.

The RNA sequencing was outsourced to Rhelixa (Japan). The initial evaluation of RNA quantity and integrity was conducted through the utilization of spectrophotometry and capillary electrophoresis. Poly(A)-selected, strand-specific libraries were constructed with the NEBNext^®^ Poly(A) mRNA Magnetic Isolation Module followed by the NEBNext^®^ Ultra™ II Directional RNA Library Prep Kit, which incorporates the dUTP method for strand specificity. The sequencing was performed on an Illumina NovaSeq 6000 or NovaSeq X Plus platform with paired-end 150 bp reads, yielding an average of approximately 4 gigabases (Gb) of data per sample, which equates to approximately 27 million reads. Transcript abundance was normalized using the transcripts per million (TPM) approach. To ensure robust transcriptomic profiling for downstream analyses, a variety of platforms were applied, including RIAS (Rhelixa Integrated Analyzers, version not specified; https://rias.rhelixa.com/rias, Rhelixa Co., Ltd., Tokyo, Japan), iDEP (ver.951, http://bioinformatics.sdstate.edu/idep95/), and RaNA-seq (version not specified; https://ranaseq.eu/)^47,48^.

### Organoid culture, passaging, and documentation of passage numbers

To ensure transparent reporting and reproducibility, we documented the passage number for every organoid experiment performed in this study. Both PDO lines were maintained across multiple passages: OICI-CS-1029 was propagated from passage 1 to passage 9, and OICI-CS-1077 from passage 1 to passage 14. For each experiment, including proliferation assays, xenograft generation, histological evaluation, WES, RNA-seq analyses, and drug sensitivity testing, the exact passage numbers used are summarized in Supplementary Table 4.

### Statistical analysis

All statistical analyses were performed using Microsoft Excel. Two-tailed Student’s t-tests were performed, and statistical significance was defined as *P* < 0.05. The study was approved by the institutional review board of the Osaka International Cancer Institute.

## Supplementary Information

Below is the link to the electronic supplementary material.

Supplementary Material 1

Supplementary Material 2

Supplementary Material 3

Supplementary Material 4

Supplementary Material 5

## Data Availability

Data presented in this study are available upon request from the corresponding author. The data are not publicly available because specific authorization was not obtained from the Institutional Ethics Committee. The RNA-seq and WES datasets generated in this study have been deposited in the DDBJ Sequence Read Archive (DRA) under the accession number JGAS000834. The datasets will be available on November 1, 2025. Until that date, the data are under embargo and not publicly accessible.https://ddbj.nig.ac.jp/search/entry/jga-study/JGAS000834https://humandbs.dbcls.jp/en/hum0401-v2.
