# Supplementary material for: Chondrosarcoma organoids reveal SHH pathway activation driven by PTCH1 and BCOR alterations
Source: Sci Rep. 2025 Dec 19;16:3239. doi: 10.1038/s41598-025-33061-z (PMC12830980; doi:10.1038/s41598-025-33061-z)

Supplementary Fig. 1. The clinical course of patients with chondrosarcoma (CS) was presented. (a) Case 1, OICI-CS-0934; A 79-year-old male patient presented with a gradually enlarging mass in the left chest wall that had been under observation for several years. Imaging revealed a lobulated bone tumor surrounding the lower rib, showing low-to-iso intensity on T1-weighted MRI and high intensity on T2-weighted MRI, measuring approximately 10 centimeters in diameter. The lesion was found to be in close proximity to the bowel, with no indications of invasion. A core needle biopsy was performed, and the diagnosis of grade 2 CS was established. Subsequent to this, a wide excision was performed, accompanied by rib resection and reconstructive surgery. A histopathological examination was conducted, which confirmed a grade 2 CS with negative margins. The patient has since remained free of recurrence or metastasis during follow-up. (b) Case 3, OICI-CS-1029; A 47-year-old female patient initially underwent curettage for a distal femoral tumor, which was diagnosed as grade 1 CS. After this, the patient exhibited local recurrence in the distal femur, necessitating extensive resection. A diagnosis of grade 2 CS was rendered. More than a decade later, she experienced another recurrence in the tibial diaphysis, confirmed as recurrent grade 2 CS, and underwent wide resection with total tibial replacement using a prosthesis. Following this, further progression was observed with recurrence in the distal fibula, for which a below-knee amputation was performed. Despite undergoing surgical intervention, the patient subsequently exhibited multiple bone metastases, including those affecting the pelvis and the thoracic spine. She underwent radiotherapy treatment for lesions affecting the pelvis and spine. However, the disease progressed, manifesting in the form of additional spinal involvement and subsequent pulmonary metastases. The patient was initiated on targeted therapy with pazopanib, but this proved ineffective, and the patient ultimately succumbed to the disease. (c) Case 4; OICI-CS-1077, A 45-year-old male patient presented with discomfort in the left hip that was initially disregarded. Thereafter, the pain in the left hip progressively worsened, and ambulation was impaired, which prompted a medical evaluation. A partial resection of a pubic bone tumor was performed due to adhesion to the bladder, and the lesion was initially diagnosed as osteochondroma. Subsequent monitoring was undertaken. Subsequent years witnessed a precipitous increase in the tumor's size, thereby prompting suspicion of malignant transformation. A core needle biopsy indicated grade 1 CS; however, a higher-grade lesion was suspected clinically. The patient subsequently underwent hemipelvectomy with visceral resection, colostomy, and urinary diversion. A pathological diagnosis of grade 2 CS was established. Despite undergoing surgical intervention, intraperitoneal recurrence occurred, which proved refractory to

radiofrequency ablation, ultimately resulting in the patient's demise due to disease progression.

Supplementary Fig. 2. The establishment of a CS PDO model, designated OICI-CS-0934. (a) The clinical images depict the CS tumors in the left rib (case 1). The coronal short tau inversion recovery (STIR) image of the magnetic resonance imaging (MRI) was presented. (b) The images of OICI-CS-0934 organoids under ALI organoid culture using phase-contrast microscopy. The morphology of organoids from both the primary culture and the xenograft-derived culture was demonstrated. (c) Tumor development was observed in NSG mice of ODX derived from the PDO of case 1. (d) Histological appearance of the original tumors and ODX of OICI-CS-0934 with hematoxylin and eosin (H&E) staining (left and middle) and Alcian Blue staining (right). STIR, short tau inversion recovery; MRI, Magnetic Resonance Imaging; ALI, air–liquid interface; ODX, organoid-derived xenograft; PDO, patient-derived organoid; NSG, NOD/SCID/IL2Rγnull; CS, chondrosarcoma

Supplementary Fig. 3. The following correlation plot illustrates the relationship between the gene expression levels of the original CS tumors and ODX in OICI-CS-1029 and OICI-CS-1077. The values displayed herein represent correlation coefficients.

Supplementary Table S1. List of primers used in the current study

Supplementary Table S2. List of all gene expression levels analyzed by RNA sequencing and normalized as transcripts per million (TPM).

Supplementary Table S3. Differentially expressed genes in CS tumors and ODX compared with normal tissues.

Supplementary Figure 1

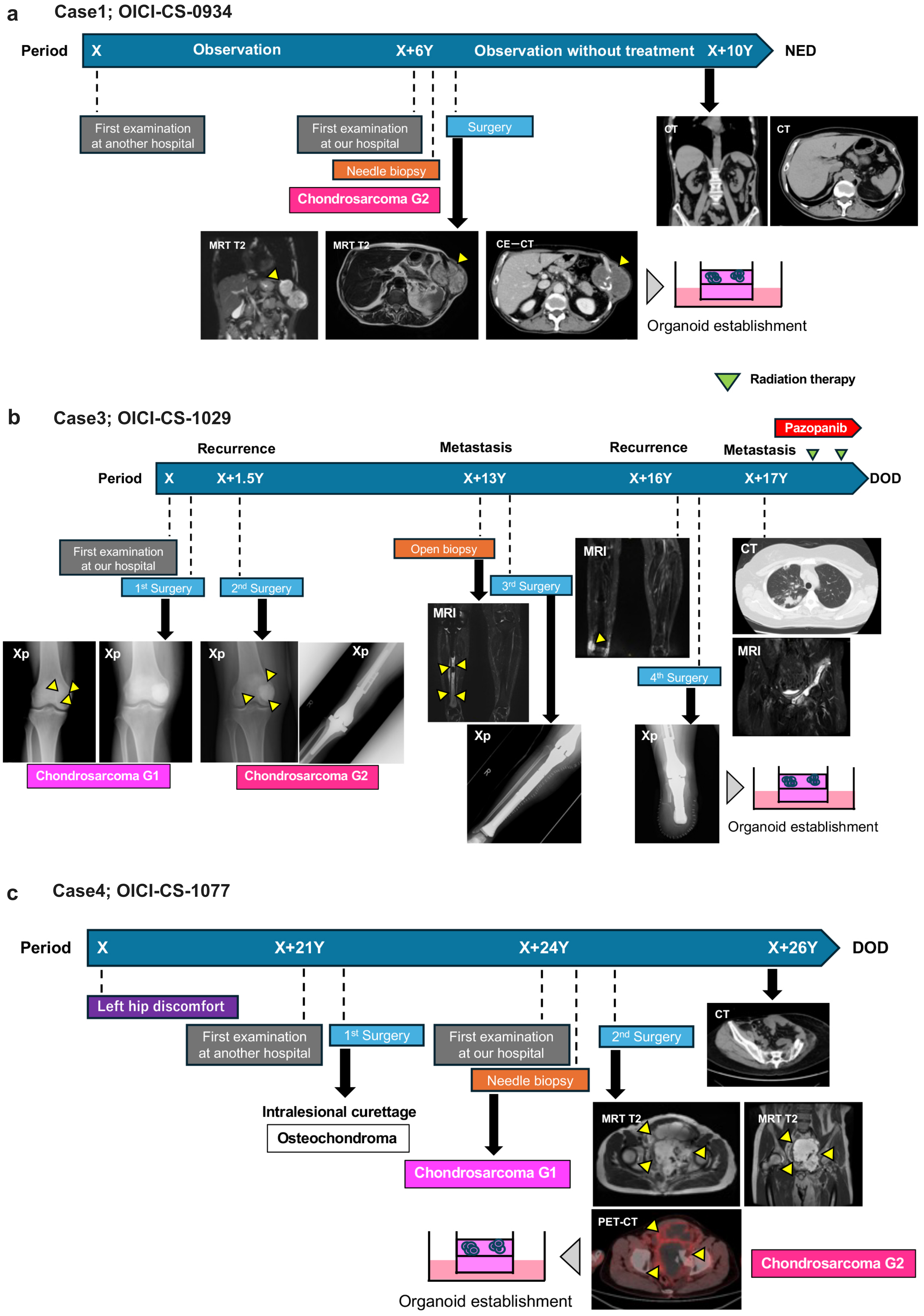

Supplementary Figure 2

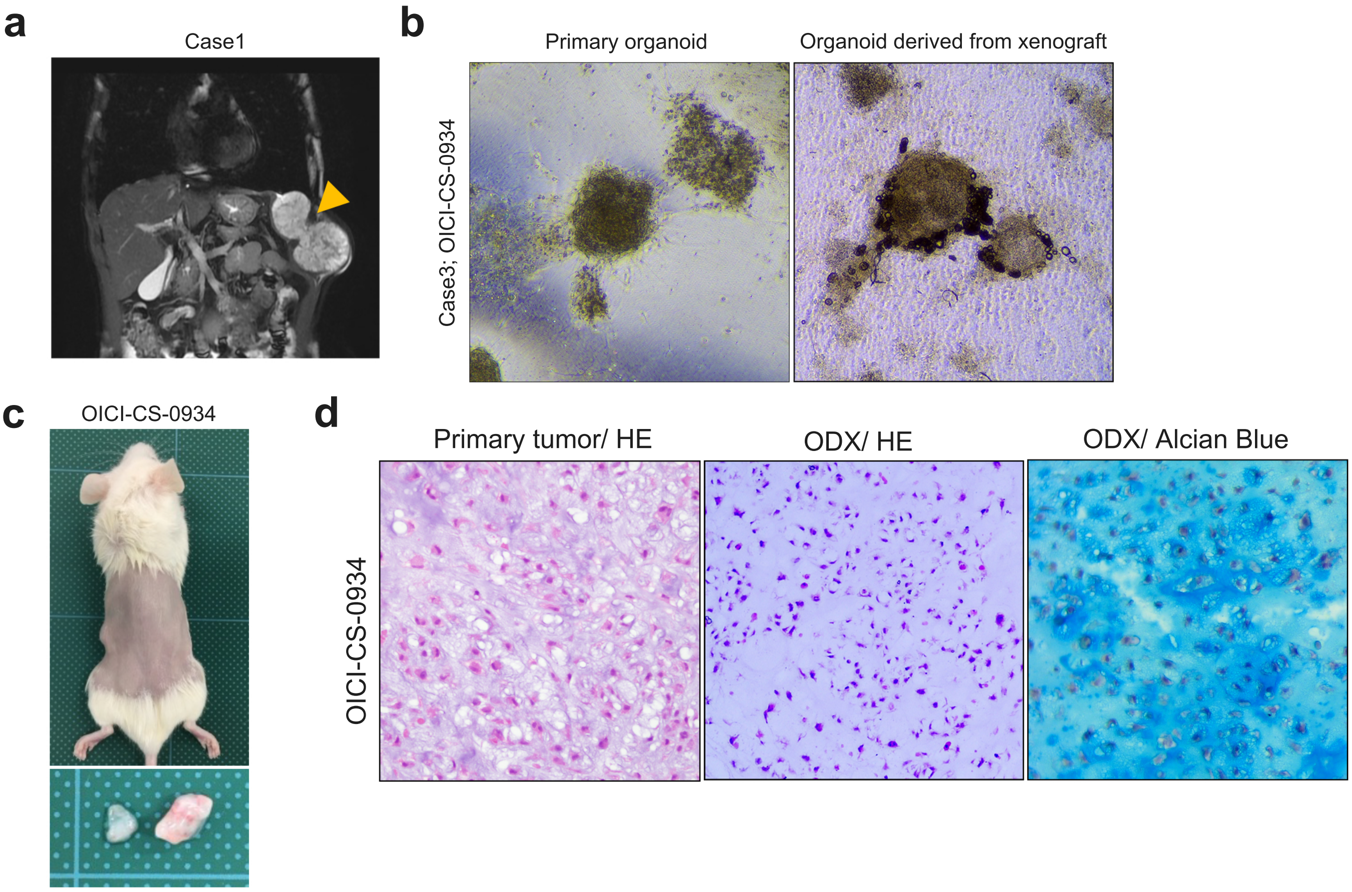

Supplementary Figure 3

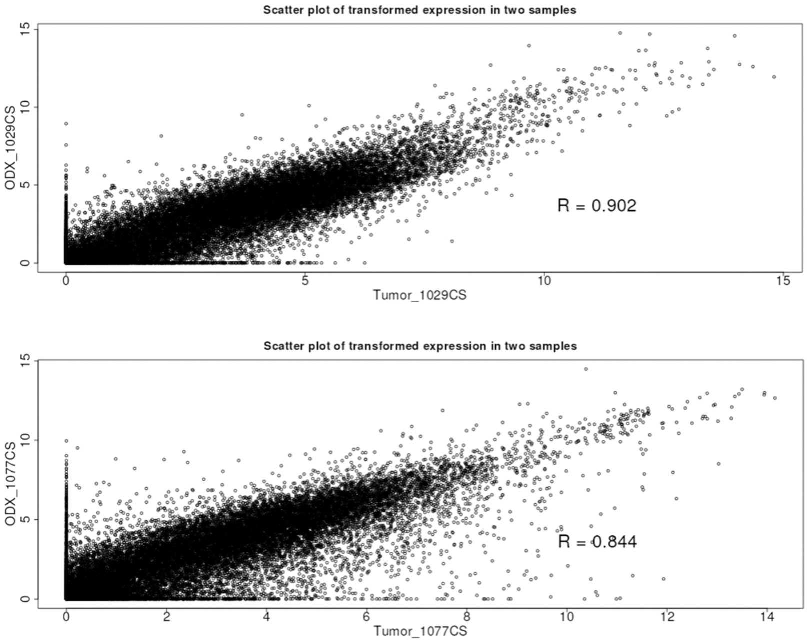

Supplement: Supplementary file 4 — Supplementary Material 4 [file 41598_2025_33061_MOESM4_ESM.pdf]
